# Notes and News

**Published:** 1889-09-28

**Authors:** 


### Notes and News.

Collected and Communicated.

Cumberland Infirmary has not much to tell in its latest report; the sudden death of the honorary secretary left the accounts in a rather unsatisfactory state; there is a debt of about £1,000. The infirmary might find it cheaper in the end to have an energetic paid secretary, though it appears rather ungrateful to say so.

A convenient little cookery book has just been issued gratis by the Liebig Company, showing the hundred and one ways in which their Extract of Meat can be used in preparation of soups, game, poultry, fish, sauces, gravies, vegetables, cold and hot meats, and invalid cookery. There are 152 capital recipes, embracing a wide range of the culinary art, and cooks and others interested in preparing the good things of the table, or nutritious food for the sick room, cannot do better than write to the Liebig's Extract of Meat Company, Limited, London, for a copy.

We may shortly expect a roar of wrath from Ruskin and other artists; for the authorities at Venice have decided that the picturesque must give way before hygienic necessities. Narrow canals, with the opposite houses almost meeting, make a romantic spectacle for tourists, who do not have to live all the year round in the dark unhealthy rooms; but we cannot wonder if the poor Venetians prefer a little more air and a little less romance. There is a medium path in all things, and in the present state of advanced sanitary engineering there is no necessity to utterly demolish any beautiful buildings in order to improve their drains. With skilful workmen, great sanitary improvements could be wrought at Venice with very little vandalism.

Montrose is to receive a munificent bequest. Provost Scott, of that town, has received the following letter from Mr. J. Willie, London: "My brother, Surgeon-General David Willie, who died at Hamburgh on 24th ult., has left, for the charities of his native town, Montrose, £2,500 to the Infirmary, and £2,500 to the public charities, to be appropriated as I may determine in consultation with the local authorities. On this latter point I shall have to request your advice, and I hope, in my absence, it may be agreeable to you to confer with my friend, Mr. F. B. Paton, of Cainbank, to whom I shall send a copy of this note. I have applied for probate of the will, and in a few weeks I hope to be in a position to pay the amounts I have named, less the Government tax. It is so long since my late brother and I left Montrose—upwards of half a century—that my name is probably unknown to you, but I feel assured that my object in addressing you will be to you a sufficient excuse." It is delightful to see how a Scotchman never forgets his native country.

Amongst the papers read before the British Association was one by Mr. R. W. Felkin, on different normal temperatures. The fact is one which often surprises a young medical man beginning to practice in the tropics. Mr. Felkin said that the normal temperature of Europeans who had resided in tropical Africa for over four years was 99·1 F.; that of Europeans who had resided for two years, 99·5 F.; that of Soudanese, 99 F.; and that of negroes living between the equator and 10 north latitude, and on the East Coast, 97·8 F.

The report of the Newcastle Hospital Sunday Fund is out, and marches with the times in showing an increase of workmen's subscriptions, and a decrease of church collections. The comparative figures as between 1887 and 1888 were as follows: The total united collections in 1887, £3,744 17s. 8d.; in 1888, £3,917 12s.; net increase, £172 14s. 4d.; collections in places of worship in 1887, £2,079 0s. 8d.; in 1888, £1,956 6s. 2d., or a decrease of £122 14s. 6d.; collections in works, 1887, £1,665 17s.; in 1888, £1,961 5s. 10d., or an increase of £295 8s. 10d. The workshops' collections were for the first time above the collections in places of worship.

Neesborough is to have its cottage hospital. It discovered that an unused meeting-house belonging to Mr. Montagu, J.P., was just what was wanted, and it hinted to Mr. Montagu that he might give it them. At an adjourned meeting of the committee of working men, a communication was received, on behalf of Mr. Montagu, not only agreeing with the project, but generously consenting to the use of the premises free of cost. The meeting was delighted at this very satisfactory consummation of the scheme, and a hearty vote of thanks was passed to Mr. Montagu for his liberality. Mr. Montagu has given directions for the premises to be altered as required, and to be in every way adapted as a cottage hospital. This will be proceeded with at once. It is arranged to have twelve beds fixed up for the accommodation of patients, and to have an experienced nurse. The public are to be appealed to for the bedding, and a ladies' committee has been formed to deal with that matter.

All sorts of letters and articles from foreign health resorts appear in our English newspapers just now, and we cannot but be surprised at times at the extraordinary "cures" that the average Briton will undergo when he has left his native country and common-sense behind him. There was one account given by a lady of a sun bath, for instance. The bathhouse is built on the side of a lake, and the water baths are supplied by two springs of pure tepid water. On the ground floor are the water baths for the ladies, and above, overhanging the water, is the sun gallery. Behind this gallery is the bathhouse for the gentlemen, and their sun gallery is on the roof of their water baths. These sun galleries are divided into a number of little cells open to the sky. A thick woollen blanket is laid on the floor and a pillow, and there the patient lies garmentless, with a linen awning shading his or her head. Forty minutes on a sunny day with cloudless sky is the prescribed time of bathing, and on clouded days from ten to twenty minutes more is added. At the end of this time the patient is rolled up tightly in the blanket like a mummy, and there remains for twenty minutes more. This is done in order to induce a profuse perspiration. At the expiration of the allotted minutes the patient is fetched, swathed in a long linen wrapper, is conducted downstairs, put into a tepid bath, and there by two stalwart attendants is scrubbed, rubbed, and sponged—water being poured over the head—for five minutes. Then follows a cold sponging of the legs and feet, and the victim is finally allowed to escape to the dressing-room.

The following vacancies are announced this week, the amount of the salary in each case being given after the name of the institution:

**Resident Medical Officers.**

East London Hospital for Children. House Physician.—Board, &c.
Northumberland County Asylum. Clinical Assistant.—Board, &c.
Flintshire Dispensary, Holywell. House Surgeon.—£100, rooms, &c.
Leicester Infirmary and Fever House. House Surgeon.—£120, board, &c.
South Devon Hospital. House Surgeon.—£100, board, &c.
London Fever Hospital. Assistant Medical Officer.—£120.
Albert Edward Infirmary, Wigan. Junior House Surgeon.—£80, board, &c.

**Non-Resident Medical Officers.**

Royal Westminster Ophthalmic Hospital. Surgeon.

---

The annual report of the Ramsgate and St. Lawrence Royal Dispensary states that the number of patients has shown an increase from 1,564 in the previous to 1,729 in this year, while the deaths have been 49 and 66 respectively, the increase in the number of deaths being mainly among children, attributable to the long-continued epidemic of measles in the spring of the year. The total expenditure was £935, and there is a balance of £91 in hand.

The proprietors of *The Christian* have in the press a handsome volume of carefully-engraved portraits, accompanied by biographic sketches of over one hundred eminent Christian men and women. The selection includes leading men of various sections of the Church, most of whom are active workers at home, and several of them zealous missionaries abroad. The book is entitled "The Christian Portrait Gallery," and will be ready in October. It will form a valuable and appropriate gift-book.

---

The Royal Victoria Hospital at Bournemouth has been informally opened, and patients now occupy the pretty wards. Little more than two years have elapsed since Her Majesty's Jubilee, and now the inhabitants have the gratification of witnessing, in the new Victoria Hospital, a practical consummation of their good wishes towards their Sovereign. At the right of the entrance hall are two tablets. On one of these the names of the life governors are inscribed in letters of gold on a slate-coloured groundwork, while another tablet, somewhat similar but smaller, records the names of the stallholders of a bazaar in Easter week this year, at which no less a sum than £2,400 was realised in aid of the institution.

---

On Thursday, the 12th inst., there was a fête in aid of the funds of the Highworth Infirmary and Convalescent Home. The grounds were opened at two, the band parading the town previously. There were many stalls, fancy and refreshment, presided over by ladies, a fishpond, fortune-telling, a shooting gallery, concerts of vocal and instrumental music, also two dramatic recitals; but the chief attraction was the "pastoral play," entitled "Quarrel of the Flowers," the young ladies being dressed in the Greek style, adorned with wreaths of flowers. The whole designing of the dresses, etc., was done by Mr. Wegrulin, the artist, who himself superintended the play. The procession walked through the grounds to the place of performance, the band playing a quick march. There were crowded audiences at each of the three performances, and it was a complete success. Over £100 was taken, which was greatly in excess of that realised last year. The tradesmen of the town gave their assistance gratis, so there were no expenses. The preliminary arrangements were admirably carried out by the matron, and the entire success of the fête was attributed to her great energy and forethought.

The Board of Management of Bradford Infirmary have issued a statement not creditable to the generosity of the folks of that town. The resources of the institution fall short of its needs by no less than £1,500 per annum. Since the opening of the new wing in 1885, the yearly expenses have increased by £2,000. To meet this, the subscriptions next year only increased by £20. In the following year there was a spurt, which added £138 per annum more. Next year there was a sad relapse, and the increase in annual subscriptions was 40s. Thus, whilst the number of beds constantly occupied on the average has risen from 113 to 178, or 30 per cent., the subscriptions have only grown by £160. This is most unsatisfactory.

---

The hunting of the germ goes on apace. At a meeting at Cupar of the Fifeshire Local Authority, General Briggs, of Strathairly, submitted a communication he had received from Prof. Chiene, Edinburgh, suggesting a scheme for a scientific investigation of the organism of pleuro-pneumonia. The professor, holding that this organism has yet to be discovered, says it will be the first step to the discovery of a vaccine for the disease. For this purpose an island such as Inchcolm would be required as an experimental station. Animals would also be needed, and if the local authority would provide these the professor is willing to give his laboratory and his assistant to do the work. One thing is very certain, that the present plan of slaughtering all animals suffering from pleuro-pneumonia is both stupid and unsuccessful, so that Prof. Chiene's plan might at least be given a trial.

---

Yet another lively meeting of governors of Suffolk General Hospital, and surely it is time this sort of thing stopped. The Rev. Prebendary Jones submitted a resolution as follows: "That a committee having been instructed at the last general meeting to revise the rules, and to report at the annual general meeting, this meeting declines to accept their report." The chairman, however, thought the first business was for someone to move that the rules be now considered according to the notice. An amendment could then be proposed. The Rev. C. H. Crossley proposed that the general board proceed to consider the rules. The chairman then put the motion, when fifteen voted in its favour and eight against. The Revs. Dr. Stantial, H. Jones, C. W. Jones, J. White, Messrs. Bland, Kaye, and Hooper then left the room, and after some remarks the meeting adjourned until the 17th October.

---

At last some honour is conferred on the Army Medical Service, and all doctors will rejoice that the bravery of Surgeon Crimmin has been rewarded by the Victoria Cross. Lieutenant Tighe, 27th Bombay Infantry (to the mounted infantry of which corps Surgeon Crimmin was attached), states that in the action near Lwekaw, Eastern Karenni, on January 1 last, four men charged with him into the midst of a large body of the enemy who were moving off from the Karen left flank, and two men fell to the ground wounded. He saw Surgeon Crimmin attending one of the men about 200 yards to the rear. Karens were round the party in every direction, and he saw several fire at Surgeon Crimmin and the wounded man. A sepoy then galloped up to Surgeon Crimmin, and the latter joined the fighting line, which then came up. Lieutenant Tighe further states that shortly afterwards they were engaged in driving the enemy from small clumps of trees, in which the Karens took shelter. Near one of these clumps he saw Surgeon Crimmin attending a wounded man. Several Karens rushed out at him. Surgeon Crimmin thrust his sword through one of them, and attacked a second. A third Karen then dropped from the fire of a sepoy, upon which the remaining Karens fled.

